# 

**Published:** 1884-12

**Authors:** 


### LIST OF SUBSCRIBERS

TO

### The Bristol Medico-Chirurgical Journal.

The attention of Subscribers is specially called to this list, in the hope that they will induce medical men who are not taking the *Journal* to support it both by subscription and by writing for it.

Names of intending Subscribers should be sent to L. M. GRIFFITHS, 9 Gordon Road, Clifton, Bristol.

The Annual Subscription (if paid in advance) is 5/-, post free.

### England.

### CHESHIRE.

MACCLESFIELD.
Sheldon, T. S.

### CORNWALL.

CALSTOCK.
Woodd, H. T.

LISKEARD.
Jenkins, J. H.

LOSTWITHIEL.
Sewart, J. H.

MEVAGISSEY.
Laing, W. A. G.

NEWQUAY.
Boyle, T.

PADSTOW.
Marley, H. F.

PENZANCE.
Bennett, W. S.

REDRUTH.
Hichens, J. S.

ST. AUSTELL.
Mason, W.

### DEVONSHIRE.

ASHBURTON.
Adams, J.

ASHTON.
Thomson, S.

BARNSTAPLE.
Harper, J.

CHILLINGTON.
Clarke, F. H.

HOLSWORTHY.
Pearce, E. T.

KINGSBRIDGE.
Webb, W. H.

LYNTON.
Berry, F. C.

BIDEFORD.
Ackland, W. H.

BRIXHAM.
Searle, G. C.

BROADCLYST.
Somer, J.

CHUDLEIGH.
Watson, J. A.

COLLUMPTON.
Potter, S. R.

DAWLISH.
Cann, F. M.

EXETER.
Blomfield, A. G.
Kempe, A. W.
Moynan, W. A.
Perkins, E. B. S.
Philipps, S. R.
Shapter, L.
Tosswill, L. H.

MORCHARD BISHOP.
Nourse, W. J. C.

PAIGNTON.
Alexander, J.

PLYMOUTH.
Clay, R. H.
Medical Society.

SALCOMBE.
Twining, A. H.

TEIGNMOUTH.
Magrath, J. A.
Workman, C. J.

TIVERTON.
Smith, J. S.

TORQUAY.
Powell, W.

WINKLEIGH.
Norman, J. H.

### DORSETSHIRE.

BOURNEMOUTH.
Fasulo, V.
Philpots, E. P.

SHERBORNE.
Williams, W. H.

WEYMOUTH.
Moorhead, J.

### ESSEX.

SOUTHEND.
Phillips, E. E.

### GLOUCESTERSHIRE.

AVONMOUTH.

Heaven, J. C.

BERKELEY.

Bridgman, I. T.

BRISTOL.

Adams, G.
Adams, J. B.
Andrews, O.
Atchley, G. F.
Baretti, T. G. L.
Baron, B. J.
Beddoe, J.
Blacker, A. E.
Blagg, A. F.
Board, E. C.
Broom, J.
Broster, A.
Burder, G. F.
Bush, J. P.
Carr, A.
Carter, W. F.
Challacombe, J. P.
Clark, T. E.
Clarke, W. M.
Coathupe, E. W.
Coe, R. W.
Coker, T. V.
Colman, T. J.
Corbould, G. G.
Cross, F. R.
Cunningham, A. G.
Dacre, J.
Davey, J. G.
Davies, D. S.
Dew, H. R.
Dobson, N. C.
Dowson, C. H.
Dowson, W.
Dunbar, E. L. W.
Edgeworth, T. F.
Elliott, C.
Elwin, J. F.
Evans, J. F.
Ewens, J.
Fendick, R. G.
Ferris and Co.
Fox, E. L.
Fyffe, W. J.
Gardiner, G.
Gibbs, A. N. G.
Giles, G. F.
Gloag, G. A.
Grace, W. G.
Griffiths, L. M.
Grote, G. W.
Harrison, A. J.
Harsant, W. H.
Hawkins, C. F.
Hayman, A. G.
Herapath, C. K. C.
Hetling, H. E.
Imlay, G. A.
Infirmary, Royal.
Isaac, G. W.
Keall, W. P.
Lansdown, F. P.
Lawrence, A. E. A.
Lawrence, H.
Laxton, H.
Lewis, J. R.
Lloyd, W. E.
Logan, F. T. B.
Luffman, W. C.
Marshall, H.
Matthews, C. E.
Mayor, T. O.
Milne, J.
Newman, C.
Newstead, J.
Nicholson, T. D.
Norton, J. A.
Norton, T. C.
Parette, J.
Parry, J. H.
Parson, T. C.
Parsons, J. D. F.
Pauli, G. C. P.
Penny, W. J.
Perkins, J. H.
Phelps, W. H.
Pickering, C. F.
Pountney, W. E.
Prichard, A.
Prichard, A. W.
Prichard, J. E.
Prowse, A. B.
Rogers, G.
Roué, W. B.
Rudge, C. K.
Shaw, J. E.
Shorland, W. E.
Skerritt, E. M.
Smith, G. M.
Smith, J. G.
Smith, R. S.
Smith, S.
Spencer, W. H.
Stansfeld, G. M.
Steele, C.
Stewart, J.
Stock, G.
Swayne, J. G.
Swayne, S. H.
Taylor, F.
Taylor, J.
Thomson, G. S.
Thurston, H. C.
Tivy, W. J.
Waldo, H.
Wallace, C. H.
Walsh, D. H.
Wathen, J. H.
Webster, T.
Welsh, F. F.
Willett, M.
Williams, E.

CHELTENHAM.

Gooding, J. C.
Newton, C. J.
Winterbotham, L.

CHIPPING SODBURY.

Grace, A.

CIRENCESTER.

Cripps, E.

DOWNEND.

Skelton, H.

DURSLEY.

Joynes, F. J.

FAIRFORD.

Cornwall, J.

FISHPONDS.

Doyne, R. W.
Mead, H. R.

GLOUCESTER.

Batten, R. W.
Bond, F. T.
Bower, E. D.
Needham, F.
Sydney-Turner, A. M.
Wilton, J. P.

HAMBROOK.

Crossman, E.
Eadon, W. F. B.

KINGSWOOD.

Grace, H.

LYDBROOK.

Fletcher, H. S.

LYDNEY.

Currie, A. S.

NORTHWOODS.

Eager, R.

STAPLE HILL.

Murray, W.

STAPLETON.

Thompson, G.

STONEHOUSE.

Watters, G. T. B.

STOW-ON-THE-WOLD.

Dening, E.

STROUD.

Cooke, A. S.
Gregory, G. S.
Howsin, E. A.
Paine, W. H.
Partridge, T.
Storry, F. W.

TETBURY.

Wickham, W.

THORNBURY.

Grace, E. M.
Salmon, W. G.
Taylor, T. H.

WESTBURY-ON-TRYM.

Ormerod, H.
Smith, G.

WINCHCOMBE.

Penruddocke, C.

WOTTON-UNDER-EDGE.

Forty, D. H.

### HEREFORDSHIRE.

HEREFORD.
Whitfeld, W. C.

ROSS.
Norman, J. W.

### LANCASHIRE.

LIVERPOOL.
Medical Institution.

MANCHESTER.
Scott, J.

POULTON-LE-FYLDE
Day, P. H.

### MIDDLESEX.

ENFIELD.
Kesteven, W. B.

LONDON.

Royal Medical and Chirurgical Society.

Jessop, C. M.

Bousfield, E. C.

Cooper, H.

Seward, W. J.

Thomson, G. J. C.

### MONMOUTHSHIRE.

ABERGAVENNY.
Steel, S. H.

CAERLEON.
Morris, J. A.

CHEPSTOW.
Lawrence, A. G.

NEWPORT.
Marsh, O. E. B.
Thomas, A. G.

PONTYPOOL.
Mason, S. B.

RHYMNEY.
Redwood, T. H.

TINTERN.
Napier, T. W. A.

TREDEGAR.
Brown, G. A.

### NORTHAMPTONSHIRE.

KETTERING.
Burges, R. E.

### SOMERSETSHIRE.

AXBRIDGE.
Smith, G.

BANWELL.
Bisdée, A. J.

BATH.
Blaxall, F. H.
Cole, T.
Cowan, F. S.
Fox, A. E. W.
Goodridge, H. F. A.
Goss, T. B.
Green, F. K.
Hopkins, H. C.
King, L.
Lowe, T. P.
Mason, F.
Russell, M. W. H.
Scott, R. J. H.
Skeate, E.
Spender, J. K.
Terry, H. G.

BRIDGWATER.
Parsons, F. J. C.
Sincock, J. B.

BRISLINGTON.
Fox, B. B.

BRUTON.
Stockwell, F.

BURNHAM.
Fraser, D. A.

CLEVEDON.
Davis, T.
Pizey, G. F. P.
Skinner, S.

COMPTON MARTIN.
Lovell, W. F.

FROME.
Burroughs, E. F. H.
Parsons, F.
Pearse, F. E.

ILMINSTER.
Hine, S. D.

KEYNSHAM.
Crisp, N.
Lodge, J.
Thomas, R. W.

LANGPORT.
Morgan, J.
Prankerd, J.

LONG ASHTON.
Fuller, J.

MIDSOMER NORTON.
Blacker, E.
Waugh, A.

MILVERTON.
Randolph, C.

MINEHEAD.
Clark, T.
Ollerhead, T. J.

NORTH PETHERTON.
Todd, W. J.

PILL.
Boys, A. H.

PORTISHEAD.
Weatherly, L. A.

SHEPTON MALLET.
Hyatt, B. N.

SOMERTON.
Valentine, E. W.

SOUTH PETHERTON.
Walter, W. H.

TAUNTON.
Alford, H. J.
Carey, J.
Liddon, W.

TEMPLE CLOUD.
Perrin, J. D.

WEDMORE.
Tyley, R. P.

WELLINGTON.
Meredith, J.
Prideaux, T. E. P.

WELLS.
Fairbanks, W.
Wade, A. L.

WESTON-SUPER-MARE.
Alford, G. E.
Alford, R.
Fraser, G. B.
Phelps, W. H. G.
Rossiter, G. F.
Roxburgh, R.
Wicksteed, F. W. S.

WINCANTON.
Colthurst, J. B.

WRINGTON.
Collins, H. W.

YATTON.
Lyons, A. de C.

YEOVIL.
Colmer, P. S. H.
Marsh, C. J.

### WILTSHIRE.

BRADFORD-ON-AVON.

Lovell, W. D.

CHIPPENHAM.

Daly, G. H.

DEVIZES.

Anstie, T. B.

MALMESBURY.

Kinneir, R.

SALISBURY.

Gowing, B. C.
Manning, H. J.
Sanctuary, T.

STRATTON ST. MARGARET.

Fernie, J.

SWINDON.

Fry, J. B.
Howse, W.
Maclean, J. C.

TROWBRIDGE.

Tayler, G. C.
Wise, N. V.

WARMINSTER.

Hinton, J.
Willcox, R. L.

WOOTTON BASSETT.

Kirkman, J. M.

### Scotland.

### EDINBURGHSHIRE.

EDINBURGH.

Pentland, Y. J.

### PERTHSHIRE.

PERTH.

Paterson, G. K. H.

### Wales.

### GLAMORGANSHIRE.

ABERDARE.

James, J. B.
Jones, E.

CARDIFF.

Fiddian, A. P.
Medical Society.
Milward, J.
Morgan, W.
Sheen, A.

MERTHYR TYDVIL.

Ward, J. L. W.

SWANSEA.

Couch, J.
Fry, J. F.
Latimer, H. A
Morgan, E. R.
Padley, G.
Rawlings, J. A.

### INDEX.

Alexander, Dr. W.—Treatment of displacements of uterus (*Review*), 132.
Amidon, Dr. R. W.—Surgical interference in affections of brain (*Extract*), 215.
Anatomical relations, The effects of certain—James Cantlie, 21.
Antipyrin, The action of (*Extract*), 284.
Arachnoid, Case of lipoma of—Dr. Braubach (*Extract*), 203.
Arteries, Twelve cases of ligature of main—J. Paul Bush, 110.
Asclepiad, The—Dr. B. W. Richardson (*Review*), 68.

Banks, W. Mitchell—Clinical notes upon surgical work (*Review*), 276.
Barnes, Dr. Robert—A synoptical guide to the study of obstetrics (*Review*), 67.
Bath waters, Guide to the—J. Douglas Kerr (*Review*), 277.
Beef tea fallacy, The (*Extract*), 70.
Biond, Dr. Domenico—Extirpation of the lung (*Extract*), 72.
Brain, Case of tumour in—J. Taylor, 128.
Brain, Changes in position of—M. J. Luys (*Extract*), 137.
Brain, Surgical interference in affections of—Dr. R. W. Amidon (*Extract*), 215.
Braubach, Dr.—Case of lipoma of arachnoid (*Extract*), 203.
Bull, Dr. E.—Operative opening of pulmonary cavities (*Extract*), 283.
Burder, Dr. G. F.,—Etiology of Catarrh, 217.
Burns, Treatment of—Dr. R. T. Morris (*Extract*), 141.
Bush, J. Paul—Twelve cases of ligature of main arteries, 110.
Buttermilk, Therapeutic properties of (*Extract*), 212.

Cancer, A new treatment of—Prof. Nussbaum (*Extract*), 70.
Cantlie, James—The effects of certain anatomical relations, 21.
Catarrh, Etiology of—Dr. G. F. Burder, 217.
Cerebral abscess, Case of—A. N. Godby Gibbs, 121.
Chorea, The nature and treatment of—Dr. E. Long Fox, 73.
Chute, H. M.—Hazeline in Menorrhagia (*Extract*), 207.
Cocaine, Muriate of (*Extract*), 280.
Collective Investigation Record (*Review*), 196.
Comingor, Dr.—Surgical treatment of sciatica (*Extract*), 137.
Copper in parasitic skin disease The oleate of—Dr. F. Weir (*Extract*), 281.
Cross, F. R.—Glaucoma, 145.
Cullingworth, Dr. C. J.—A short manual for monthly nurses (*Review*), 68.
Currie, Dr. A. S.—Notes of cases of diphtheritic paralysis, 186.

Diaphragmatic (sub-) abscess, Case of—J. Harper, 184.
Diphtheria, Case of—Dr. R. Shingleton Smith, 61.
Diphtheritic paralysis, Notes of cases of—Dr. A. S. Currie, 186.
Doran, Alban H. G.—Tumours of ovary &c. (*Review*), 279.

Elliott, Dr. C.—A case of laryngeal phthisis, 260.

Eye, Diseases and injuries of—Dr. L. H. Tosswill (*Review*), 63.

Fat embolism—Dr. Park (*Extract*), 208.
Fat embolism, Case of—A. W. Prichard, 54.
Ferris and Co.—Notes on new remedies (*Review*), 69.
Fischer, Dr.—Antiseptic sugar dressings for wounds (*Extract*), 71.
Flat-foot, and its cure by operation—Prof. A. Ogston, 1.
Fournier, Dr.—Hereditary cerebral syphilis (*Extract*), 139.
Fox, Dr. E. Long—Case of spina bifida, 45; the nature and treatment of chorea, 73.
Fyffe, Dr. W. J.—Abscess of liver, 245.

Gastric ulcer, Case of—Dr. J. E. Shaw, 42; Dr. W. H. Spencer, 41.
Germ theory and heroic doses (*Extract*), 288.
Gibbs, A. N. Godby—Case of cerebral abscess, 121.
Gibier, Paul—Rabies in birds (*Extract*), 204.
Glaucoma—F. R. Cross, 145.
Goitre, Iodine and Ergotine in (*Extract*), 205.
Gonorrhœa, The treatment of—Dr. S. C. Gordon (*Extract*), 143.
Gordon, Dr. S. C.—The treatment of gonorrhœa (*Extract*), 143.

æmophilia, Two cases of—J. Greig Smith, 264.
Hamamelis Virginica, Pharmacology of (*Extract*), 204.
Harper, J.—Case of sub-diaphragmatic abscess, 184.
Harrison, Reginald—Observations on lithotomy, &c. (*Review*), 66.
Hazeline in Menorrhagia—H. M. Chute (*Extract*), 207.
Heart, Case of rupture of right auricle of—Dr. George Thompson, 48.
Henry, Dr. Louis—Massage (*Extract*), 287.

Insane, General paralysis of—Dr. T. S. Sheldon, 235.
Intestinal obstruction, Three cases of—Dr. A. Sheen, 167.
Intra-venous injection of saline solutions—Dr. Jennings (*Extract*), 72.

Jennings, Dr.—Intra-venous injection of saline solutions (*Extract*), 72.
Jequirity—Dr. S. Pollak (*Extract*), 286.

Kairin, Physiological action of—M. Loych (*Extract*), 139.
Keall, W. P.—Case of suturing of olecranon, 50.
Keetley, C. B.—An index of surgery (*Review*), 134.
Kerr, J. Douglas—Guide to the Bath waters (*Review*), 277.

Leamington waters, The—Dr. F. W. Smith (*Review*), 135.
Lithotomy, &c., Observations on—Reginald Harrison (*Review*), 66.
Liver, Abscess of—Dr. W. J. Fyffe, 245.
Loych, M.—Physiological action of kairin (*Extract*), 139.
Lung, Extirpation of the—Dr. Domenico Biond (*Extract*,) 72.
Luys, M. J.—Changes in position of brain (*Extract*), 137.

Malignant pustule, Case of—Dr. L. A. Weatherly, 124.
Malignant pustule communicated by a fly—M. Mollière (*Extract*), 138.
Massage—Dr. Louis Henry (*Extract*), 287.
Materia medica and therapeutics—J. Mitchell Bruce (*Review*), 201.
Medical Chronicle, The—(*Review*), 278.

Medical fashions in the nineteenth century—Dr. E. T. Tibbits (*Review*), 64.
Moeller, M.—The action of respirators, 136.
Mollière, M.—Malignant pustule communicated by a fly (*Extract*), 138.
Morris, Dr. R. T.—The treatment of burns (*Extract*), 141.

New York Academy of Medicine, Transactions of (*Review*), 278.
Nurses, A short manual for monthly—Dr. C. J. Cullingworth (*Review*), 68.
Nussbaum, Prof.—A new treatment of cancer (*Extract*), 70.

Obstetrics, A synoptical guide to the study of—Dr. Robert Barnes (*Review*), 67.
Ogston, Prof. A.—Flat-foot, and its cure by operation, 1.
Oleate of zinc (*Extract*), 209.
Oliver, Dr. Geo.—On bedside urine testing (*Review*), 67.
Ovary, Tumours of, &c.—Alban H. G. Doran (*Review*), 279.
Ozœna, Treatment of (*Extract*), 213.

Park, Dr.—Fat embolism (*Extract*), 208.
Peptonised food, The use of—Dr. W. H. Spencer, 32.
Phthisis, A case of laryngeal—Dr. C. Elliott, 260.
Phthisis, Contagiousness of (*Extract*), 210.
Phthisis, Picrotoxin in (*Extract*), 210.
Physiological physics, The elements of—J. McG. Robertson (*Review*), 275.
Picrotoxin in phthisis (*Extract*), 210.
Poliomyelitis anterior, Case of sub-acute—Dr. R. Shingleton Smith, 175.
Pollak, Dr. S.—Jequirity (*Extract*), 286.
Prichard, A. W.—Case of fat embolism, 54.
Pulmonary cavities, Operative opening of—Dr. E. Bull (*Extract*), 283.

Rabies in birds—Paul Gibier (*Extract*), 204.
Rabies, Prevention of (*Extract*), 205.
Remedies, Notes on new—Ferris and Co. (*Review*), 69.
Respirators, The action of—M. Moeller (*Extract*), 136.
Richardson, Dr. B. W.—The Asclepiad (*Review*), 68.
Robertson, Dr. John—Surgical delusions (*Extract*), 144.
Robertson, J. McG.—The elements of physiological physics (*Review*), 275.
Roué, Dr. W. B.—Case of sudden death from asphyxia in phthisis, 181.

Scarlet Fever, The after treatment—G. Smith, 263.
Sciatica, Surgical treatment of—Dr. Comingor (*Extract*), 137.
Sclerosis of the spinal cord, Lateral—Dr. J. E. Shaw, 270.
Shaw, Dr. J. E.—Case of gastric ulcer, 42; lateral sclerosis of the spinal cord, 270.
Sheen, Dr. A.—Three cases of intestinal obstruction, 167.
Sheldon, Dr. T. S.—General paralysis of insane, 235.
Sims, The late Dr. J. Marion (*Extract*), 70.
Smith, Dr. F. W.—The Leamington waters (*Review*), 135.
Smith, Dr. R. Shingleton—Case of diphtheria, 61; case of sub-acute poliomyelitis anterior, 174.
Smith, J. Greig—Ingrowing toe-nail, 99; stricture of urethra, 154; two cases of hæmophilia, 264.
Smith, G.—The after-treatment of scarlet fever, 263.
Spencer, Dr. W. H.—Case of gastric ulcer, 41; the use of peptonised food, 32; Thomsen's disease, 159.

Spina bifida, Case of—Dr. E. Long Fox, 45.
Statistics, The curse of (*Extract*), 213.
Sudden death from asphyxia in phthisis, Case of—Dr. W. B. Roué, 181.
Sugar dressings for wounds, Antiseptic—Dr. Fischer (*Extract*), 71.
Surgery, An index of—C. B. Keetley (*Review*), 134.
Surgical delusions — Dr. John Robertson (*Extract*), 144.
Surgical follies (*Extract*), 210.
Surgical work, Clinical notes upon—W. Mitchell Banks (*Review*), 276.
Suturing of olecranon, Case of—W. P. Keall, 50.
Syphilis and pseudo-syphilis—A. Cooper (*Review*), 199.
Syphilis, Hereditary cerebral—Dr. Fournier (*Extract*), 139.

Taylor, J.—Case of tumour in brain, 128.
Tetanus following hypodermic injection of quinine (*Extract*), 285.
Tholozan, M.—Excision of uvula (*Extract*), 138.
Thompson, Dr. George — Case of rupture of right auricle of heart, 48.
Thomsen's disease—Dr. W. H. Spencer, 159.
Toe-nail, Ingrowing — J. Greig Smith, 99.
Tosswill, Dr. L. H.—Diseases and injuries of eye (*Review*), 63.
Tubercle of ascending frontal convolution (*Extract*), 204.

Urethra, Stricture of—J. Greig Smith, 154.
Urine testing, On bedside—Dr. Geo. Oliver (*Review*), 67.
Uterus, Treatment of displacements of — Dr. W. Alexander (*Review*), 132.
Uvula, Excision of—M. Tholozan (*Extract*), 138.

Weatherly, Dr. L. A.—Case of malignant pustule, 124.
Weir, Dr. F. — The oleate of copper in parasitic skin diseases (*Extract*), 281.

J. W. ARROWSMITH, PRINTER, QUAY STREET, BRISTOL.

